# Medical and Physical Intelligence

**Published:** 1821-04

**Authors:** 


### Medical and Physical Intelligence.

A BOY born, at Morillon, October the 20th, 1817, and manifesting now the signs of puberty, was presented at a late sitting of the Society of the College of Physicians of Paris. He is fat and strong, and weighs about sixty-seven pounds; his height is three feet two inches. The circumference of his head is one foot seven inches; that of the thorax, immediately below the arm-pits, two feet; the diameter of the pelvis, from the pubis to the sacrum, five inches and nearly a half. The length of the penis, in a lax state, from the pubis to the end of the glans, is three inches and seven lines; its diameter at the base, three inches five lines; when in the state of erection, the penis is five inches in length: the testicles are not quite proportionally large. This boy, Jaques-Aimé Savin, has abundant, shining, chesnut-coloured, hair, which is somewhat stiff and curly. The body generally is slightly covered with hair, of a lighter colour than that on the head. There is a growth of downy hair, of the colour of that on the head, in the ordinary situation of the beard and whiskers; about the pubis, the hair is as abundant as it commonly is at the age of sixteen or eighteen years. The skin generally is rugous, roughish, and resistant to the touch. His *digestion* is very active, and he eats with voracity not the most delicate sorts of food. His muscles are well-marked, and his movements are firm. The strength of his loins was found to be equivalent to the weight of 90 pounds, and that of his wrist 27.* He can lift and carry from one place to another a weight of from forty to fifty pounds. He is bold and courageous; he wrestles with persons much older, and even bigger, than himself, and despises any trials of the kind with little boys. Fighting and wrestling are his favourite amusements. His voice is strong, and resembles in its timbre that of a lad of sixteen or eighteen. His external organs of sense present nothing particular. His intellectual faculties are not developed proportionately to his other functions, and especially not to that of his genital organs. His memory is particularly good; but his judgment is only conformable with his age and education,† (speaking in relation to persons in general;) and the same may be said in respect to his imagination. We shall transcribe what Dr. Breschet, whose description we are here abridging, says of his *passions*, in his own words, omitting only one short passage: "Les organes genitaux ne restent pas dans un état d'inertie. Souvent le penis entre en erection, et la presence de jeunes filles ou de femmes produit cet effet. Dans ces circonstances, toute la personne de Savin est animée et agitée; les yeux, la parole, et le geste sont en harmonie, et par un

* The strength of the loins of an adult man is said to be commonly about 290 pounds, and that of the wrist 110, when employed in the following way:—To try the former part, the *dynamometer* is held by the feet, whilst the hands pull at a bar placed horizontally; for the latter, a cushion, sustained by a bar which communicates with the spring and needle of the dynamometer, is struck by the fist.

† His father is a baker.

instinct particulier il cherche à porter ses mains vers les organs genitaux d'un sexe different du sien, sans trop savoir les fonctions auxquelles sont appellés et les uns et les autres; il parait certain que . . . . . . . . . . *ni* la copulation ne sont connues de cet enfant."

He presented nothing extraordinary, with respect to size or weight, at his birth: the midwife, however, told the parents that his bones seemed to be larger than ordinary; the sutures of the cranium appeared, also, to be firmer than they commonly are at the time of birth. He was suckled by his mother for eleven months, and was weaned without experiencing any indisposition. About this epoch it was remarked, accidentally, that he had a severe inflammation about the penis; and a medical practitioner, who was called to see him, found that he had severe paraphymosis. Emollient anodyne fomentations and poultices dissipated this inflammation nearly, but not entirely, in the space of two months. The cutting of his teeth was precocious: at the age of three months, the first incisors appeared in the upper jaw; at four months, seven teeth were to be seen; and at one year, there were twenty. Since this epoch he has not cut any.

His parents are healthy, and present nothing remarkable in their organization. His father, twenty-five years of age, is of a lymphatico-sanguineous temperament, five feet two inches in height, of a slender habit, and arrived at puberty at the age of fourteen. His mother is twenty-seven years of age, of a sanguineous temperament, of a weak habit, menstruated at fifteen, was married at twenty-four, and at the lapse of fourteen months from her marriage, the boy above described was born.

---

An account has been lately read to the Society above named, of what the narrator—Dr. Charpentier, physician to the royal forces of the marine, at Guerigny, near Nevers,—considers to have been instances of spontaneous combustion of the human body. We shall give the history in detail, because there are some circumstances in it that seem very forcibly to favour the inference above mentioned; whilst the occurrence of the phenomenon in two persons at the same time, on the contrary, furnishes grounds for a strong suspicion that the combustion arose from an external source.

" On the 15th of January, 1820, at ten o'clock in the evening, several neighbours of Mrs. P—, of Nevers, perceived a peculiar odour, which they thought similar to that of broiled animal matter and burning wool, only more disagreeable and nauseous. They saw neither smoke nor vapour issue from any of the adjacent houses; and at last, agreeing amongst themselves that this odour was produced by the burning of the remains of an old Carmelite nun, who had died in the neighbourhood that day, they retired to bed without making any further inquiries.

On the 13th, in the morning, a woman, living near the place, who had a second key to the door of the house, because she was in the habit of going there daily to assist the servant in attending on her mistress, opened the door to go and perform her ordinary duties. On entering the room,* a dense vapour issued out, accompanied with an insupportable stench, that almost suffocated her. She retreated from the house, crying out in the most violent manner for help. The neighbours came about her; and, after waiting a few moments to let the vapour escape, they proceeded to examine the state of the room. They found neither Mrs. P— nor her servant. At first they saw no appearance of dead bodies, but they immediately recognized that Mrs. P.'s bed was entirely burned. Its different parts, however, preserved their form; but, on the slightest touch, it all sunk away, and the bedstead, pallaisse, mattress, feather-bed, sheets, blankets, and curtains,† were reduced to a cinder. Before they stirred these cinders they examined the fire-place, in which they found no wood, nor any charcoal, in combustion: the fire had not been covered, and it had probably gone out for want of wood. A candlestick stood in the fire-place, and another, on the ground, in the middle of the room; there was no candle in either of them.‡

On proceeding to examine the ashes, or remains of the combustion, there was found, in front of the spot which had been occupied by the bed, the extremity of a leg covered by a stocking, with a shoe on the foot, and which was recognized to be part of the right leg of the servant. It was the only portion of the body of this woman that had not been reduced to ashes.§

The cranium of the mistress, devoid of the scalp, which had been burned, was found in a situation corresponding with that in which the head would be as the woman lay in bed. This was the only portion of her body that had not been utterly destroyed by combustion, excepting a small fragment of the neck, or rather the skin of the neck, that had been enveloped in a red kerchief, which had probably served as a cravat, and of which there were yet some remains immediately attached to the preserved portion of the neck.

The servant's bed, which was very near to that of the mistress, was untouched, as well as the table, chairs, and other furniture of the room, excepting a wooden clock, hung up against the wall beside the bed,‖ that, having preserved its form, fell into ashes on the first movement.

Although the room had no ceiling, the beams and rafters, which

---

* From the tenor of this expression, and the want of notice of any other part of the house, there is reason for inferring that the house of Mrs. P. consisted of but a single room, the door of which opened into the street. It is not at all rare in France for persons having as much property as Mrs. P. had, to live in a house so constructed.—Editor.

† "The curtains were woollen."

‡ "A lighted candle had probably been placed in that situate in the middle of the room, and had been entirely consumed."

§ "From the position of this leg, it was considered that the servant died lying across her mistress, with the right foot resting on the ground, and the left leg, as well as the rest of the body, on the bed. It was presumed that the servant had fallen in that position as she went to help her mistress."

‖ It is not possible to discern, from the original account, which bed is meant: we have translated literally, and have preserved the narrator's distinction of paragraphs. From the servant's bed having been just spoken of, in the same paragraph, we have some reason for suspecting that is signified.—Editor.

were very near to the top of the bed, were not burned, but they were black and felt very hot. All the things about the room, especially such as were close to the bed, were extremely humid; which was owing, without doubt, to condensation of the dense vapours with which the room was filled on being first entered.

As there were no other persons in the house than these two women, and the accident not having been discovered until the ensuing morning, no one can possibly know the cause of it.

During the night of the 12-13th of January, (that of the accident,) the weather was very serene, the air dry, and the cold very severe, since the thermometer marked ten degrees below zero.*

The mistress was ninety years old; the servant sixty-six; they were both of a weak constitution, thin, and meagre; their food was but bad, although the mistress had an income of 6000 francs. She, for some time past, had drunk *eau de Cologne* to great excess. It is said, however, that for two years past, from the remonstrance and advice of her physician, she had taken it in a less quantity, which was indispensable for the support of her declining strength, especially as she had eaten hardly any thing since this habitual abuse of the eau de Cologne. The servant also ate but little; she now and then took a little brandy; but her nourishment consisted chiefly in good old wine, hot and well sugared. She often took this in sufficient quantities to make her tipsy. It is believed that the excessive cold of the night in question had led her to drink to excess.†

---

*The Société Medicale d'Emulation* proposes the following as a prize question: the memoirs, written in French or Latin, are to be sent, before the 31st of August, 1822, to the Secretaire-générale, Rue Bertin-Poirée, No. 10, at Paris. The value of the prize is 500 francs.

"What are the dispositions and structure of the system of organs called the *nervous ganglions of the organic life*, *sympathetic nerve*, *great intercostal*, *trisplanchnique*, &c.?

"What are the functions of this system of organs?

"And, as far as is known, what are the diseases in which it is essentially affected?"

The Society proposed this question for the concourse in 1819. Two memoirs arrived: one of them approached the desired object; but the author of this memoir having made himself known before a judgment could be pronounced on it, and having besides withdrawn his memoir, the Society, to which this question appears too important to be thus abandoned, proposes it for another concourse. It requires that the candidates direct their efforts to replies to the three points of the question, founded on dissections made of human subjects and diverse animals, and on experiments and clinical observations: it is a memoir constituted of positive facts that is desired.

---

* Equivalent to 14° Fahrenheit.

† Dr. C. produces a long discussion on the circumstances above narrated, with the view of supporting the inference already stated; but we have not thought it necessary to transcribe it.

We promised, in the last Number, to give an analysis of the contents of Dr. Magendie's Journal of Experimental Physiology: want of space for it, however, obliges us to postpone it. The paper next in regard to its interest, for the purposes of practice, to that on *rabies*, is one on *a new extract of opium*, by Mr. Robiquet. We have just space enough for an abstract of this, on the present occasion.

It has been ascertained that the most active properties of opium reside in two substances which may be obtained in distinct forms, and which produce very different effects: *narcotine*, and *morphine*, as they have been named. The former is a very powerful irritant to the nervous system, as it appears from the experiments of Dr. Magendie, and it seems to be this substance which produces the effects which we so much wish to avoid in the administration of this medicine, and which are obviated, to a certain extent, by giving it in the form of the *black-drop*, or other analogous acid preparations. *Morphine*, according to the evidence of the same experiments, and others made on human beings, produces sedative effects without symptoms of the slightest irritation. To separate the narcotine, then, from the other parts of opium, is a very desirable object of pharmacy, and Mr. Robiquet says it may be easily effected in the following way:

"I macerate," says Mr. Robiquet, "common opium, divided into small pieces, in cold water, as it were for the aqueous extract of opium; I filter the solution, evaporate it to the consistence of a thick syrup, and treat it, in convenient vessels, with rectified ether; the whole is then shaken a great many times, before the ethereous tincture is poured off; this, being separated, is then submitted to distillation, that the ether may be drawn from it. This process is repeated as often as crystals of *narcotine* are obtained as a residue of the distillation. When the ether is without action, I evaporate the solution of opium to a consistence proper for pills; and I obtain, by this means, an extract wholly exempt from narcotine."

Dr. Magendie says he has employed this extract in practice for some time, and that he has hitherto seen only advantageous results from it, even in persons who derived no benefit from the ordinary aqueous extract.

As this account may, we suspect, not be perfectly perspicuous to such of our readers as are not versed in chemistry, we shall add a few explanatory observations. Ether, when added to the *thick aqueous extract*, dissolves the *narcotine*, whilst it has no action on the *morphine:* the latter is thus left behind in the aqueous extract when the ethereous liquid is decanted; and fresh ether is added to the aqueous extract, and poured off, (after having been well shaken with it,) as the former, as long as it is found that ether, so mixed with the aqueous extract, leaves behind no crystals of narcotine on being submitted to distillation. The aqueous solution left after this treatment contains the morphine, and has only to be evaporated to a proper consistence to be fit for medical purposes.

### REPORT OF DISEASES.

WE omitted, in the last two Numbers of this Journal, to make any report of the prevailing diseases, because no new circumstance worthy of remark had occurred, and we were unwilling to draw upon the patience of our readers by ringing perpetual changes upon rheumatism and winter-cough —winter-cough and rheumatism. From this dilemma we have been relieved by the March winds, and find, upon consulting our register, the more interesting names of gastritis, enteritis, and pleuritis. Now, although we would not be understood as enumerating either of the former among *prevailing* diseases, still the occurrence of individual cases which we have seen, if viewed in relation to the inflammatory affections of the chest, tend to illustrate the nature of the change which has taken place in the prevalent form of disease. This, which for some months had been chronic in its nature, having generally the mucous structures for its seat, has now become acute, attacking principally the serous membranes. This last remark, which in a general point of view applies to adults, does not hold good with regard to children; among whom inflammation, though much more acute than before, has almost invariably been seated in the mucous membrane of the lungs: indeed, acute bronchitis has prevailed among children like an epidemic, frequently coming on with so much severity as to render the most active remedies indispensible, and in some cases unavailable.

We had formerly occasion to allude to the prevalence of erysipelas; a considerable number of instances have continued to present themselves, and the disease has in general been more than usually severe. Indeed, during the last few months it has committed considerable ravages, and the occurrence of severe, and even fatal, cases has not been confined to London; many having taken place in military hospitals, notwithstanding the advantages of those over our civil institutions in this town, from purer air and disciplined regulations. It is singular, too, that it has prevailed to some extent in one regiment of Guards stationed at the Tower; while those in the west end of the City have been comparatively free from it. This would prove, if additional proof were required, the strong disposition evinced by erysipelas to occur as an endemic in particular situations. But some recent observations have led us to suspect that it is really contagious: amongst other reasons for this inference, we have known the same individual attacked two several times with erysipelas, during attendance upon a person labouring under that disease;—a coincidence so remarkable, even though it were singular, would point out this subject as worthy of further investigation.

During the early part of the month, when the weather was comparatively mild, scarlet fever, in children especially, began to appear in an epidemic character: we saw, in the same range of practice, nearly twenty cases in about a week, at that time; but not a single instance within the last fortnight. Scarlet fever was then taking the lead of bronchitis, as regards children, and whooping-cough was becoming rare: the latter affections have again resumed their former prevalence. The history of whooping-cough, in a general point of view, has been this:—Symptoms of bronchitis, without whooping, for about eight or ten days; then whooping, with less of inflammation; and, in about another week, or somewhat less, signs of great disturbance of the nervous system; and, when these are particularly severe, the case will terminate in death, if not well treated. The constant, and most remarkable, appearances on dissection, in these cases, are signs of inflammation about the medulla oblongata and upper part of the spinal marrow; and, almost constantly, (evidence of some degree of inflammation having been seated here, has never been wanting, though the signs of it have been more or less dissipated,) signs of inflammation, of no inconsiderable intensity, in the mucous membrane of the lower part of the trachea,—the situation corresponding with that of the *inferior glottis* of birds, (we mean to offer a hint here to comparative physiologists,)—and about the bifurcation of the bronchia. We shall say more on this point at a future time.

Small-pox exists in several parts of the northern and western parts of London; and the unprotected subjects for it are not few amongst the poorer classes of the people.

### METEOROLOGICAL JOURNAL.

By Messrs. William Harris and Co. 50, Holborn, London.

*From February* 20 *to March* 19, *inclusive.*

| Day of Month. | Moon. | Rain gauge | THERM. | | | BAROM. | | DeLuc's Hygro. | | WIND. | | ATMOSPHERIC VARIATION. | | |
|---|---|---|---|---|---|---|---|---|---|---|---|---|---|---|
| Feb. | | | | | | | | | | | | | | |
| 20 | | | 33 | 37 | 33 | 30·26 | 30·15 | 57 | 58 | WNW | W | Cloudy | Fine | Rain |
| 21 | | ·03 | 36 | 40 | 33 | 30·20 | 30·24 | 60 | 60 | NW | N | Cloudy | Fine | Cloud. |
| 22 | | | 35 | 39 | 31 | 30·31 | 30 33 | 59 | 57 | NNE | W | Cloudy | Fine | Cloud. |
| 23 | | | 35 | 39 | 34 | 30·29 | 30·20 | 58 | 58 | WSW | W | Cloudy | | |
| 24 | | | 36 | 40 | 33 | 30·15 | 30·12 | 60 | 59 | E | ENE | Fog | Cloud. | |
| 25 | ☽ | | 35 | 42 | 34 | 30·09 | 30·07 | 59 | 59 | NE | NE | Fog | Cloud. | |
| 26 | | | 36 | 38 | 26 | 30·08 | 30·07 | 58 | 59 | E | ENE | Cloudy | | |
| 27 | | | 29 | 31 | 28 | 29·87 | 29·64 | 57 | 59 | S | E | Fine | | |
| 28 | | ·10 | 29 | 31 | 31 | 30·32 | 30·30 | 58 | 58 | E | ENE | Snow | | |
| Mar | | | | | | | | | | | | | | |
| 1 | | | 33 | 40 | 38 | 29·37 | 29·64 | 60 | 64 | SE | S | Rain | Cloud. | Cloud. |
| 2 | | | 42 | 49 | 46 | 29.87 | 29·82 | 63 | 67 | S | S | Fine | Rain | |
| 3 | | | 49 | 55 | 48 | 29·77 | 29·67 | 69 | 71 | SW | SW | Rain | | Cloud. |
| 4 | ● | ·53 | 49 | 51 | 37 | 29·63 | 29·70 | 68 | 67 | SW | W | Rain | Cloud. | |
| 5 | | | 38 | 40 | 33 | 30·96 | 30·02 | 65 | 61 | NNE | SW | Cloudy | | |
| 6 | | | 36 | 39 | 34 | 29·87 | 29·50 | 59 | 60 | SSE | SSW | Cloudy | Rain | |
| 7 | | ·29 | 36 | 40 | 38 | 29·60 | 29·53 | 61 | 65 | W | W | Cloudy | Rain | |
| 8 | | ·28 | 41 | 43 | 39 | 29·39 | 29·31 | 63 | 63 | W | WSW | Fine | | Rain |
| 9 | | ·05 | 45 | 56 | 48 | 29·40 | 29·63 | 60 | 62 | SW | SW | Fine | Sho'ry | Rain |
| 10 | ☾ | ·10 | 49 | 55 | 46 | 29·69 | 29·75 | 63 | 60 | SW | SW | Fine | Hail | Fine |
| 11 | | | 47 | 54 | 42 | 29·85 | 29·44 | 62 | 61 | WSW | SSW | Fine | | |
| 12 | | | 43 | 51 | 44 | 29·96 | 30·01 | 60 | 59 | SW | W | Cloudy | Fine | Rain |
| 13 | | | 45 | 57 | 40 | 30·03 | 30·03 | 59 | 59 | SW | W | Fine | | |
| 14 | | ·05 | 43 | 53 | 37 | 30·08 | 30·32 | 60 | 55 | NNE | NE | Fine | | |
| 15 | | | 40 | 51 | 37 | 30·36 | 30·36 | 51 | 52 | SSW | S | Cloudy | Fine | |
| 16 | | | 39 | 53 | 39 | 30·30 | 30·20 | 56 | 55 | E | S | Fog | Fine | |
| 17 | | | 41 | 55 | 39 | 29·91 | 29·72 | 51 | 55 | SW | SW | Cloudy | Fine | |
| 18 | ○ | | 42 | 59 | 34 | 29·57 | 29·44 | 54 | 56 | WNW | WNW | Fine | Stormy | Cloud. |
| 19 | | ·20 | 39 | 51 | 37 | 29·25 | 29·31 | 55 | 53 | NW | NW | Fine | | |

The quantity of rain fallen in the month of February, is 13-100ths of an inch.

